# Optimizing control parameters for Huanglongbing disease in citrus orchards using SAIR-SI compartmental model, epidemic final size, and genetic algorithms

**DOI:** 10.1007/s00285-024-02161-1

**Published:** 2024-12-13

**Authors:** Andrés Anzo Hernández, Uvencio José Giménez Mujica, Carlos Arturo Hernández Gracidas, José Jacobo Oliveros Oliveros

**Affiliations:** 1 CONAHCYT- Investigadoras e Investigadores por México, Facultad de Ciencias Físico-Matemáticas, Benemérita Universidad Autónoma de Puebla, Avenida San Claudio y 18 Sur, Colonia San Manuel, 72570 Puebla, Puebla México; 2 Facultad de Ciencias Físico-Matemáticas, Benemérita Universidad Autónoma de Puebla, Avenida San Claudio y 18 Sur, Colonia San Manuel, 72570 Puebla, Puebla México; 3 Área de Matemáticas Aplicadas, Centro de Investigación en Matemáticas, A.C., De Jalisco s/n, Col. Valenciana, 36023 Guanajuato, Guanajuato México

**Keywords:** Huanglongbing disease, Compartment SAIR−SI model, Epidemic final size, Evolutionary algorithm, 92-10

## Abstract

Huanglongbing (HLB) is a bacterial disease that affects citrus trees worldwide. We present an innovative approach for identifying optimal control and risk measures for HLB in citrus orchards. Our method is based on a mathematical model that incorporates the number of roguing trees and a logistic growth model for the dynamic of the Asian Citrus Psyllid (ACP), the primary vector for HLB transmission. We derive an expression for: (1) the basic reproduction number $R_{0}$; (2) the final size for the number of roguing trees; and (3) the transmission risk. The above let us propose a difference map equation that assesses this final size with a low computational cost. We use this difference map in an evolutionary algorithm to identify the most effective combination of control parameter values for reducing HLB transmission, including the timing and frequency of roguing and the use of insecticides. In this sense, we propose two control strategies, which we called tree-centered and vector-centered.

## Introduction

Citrus greening disease, also known as Huanglongbing (HLB), meaning “*yellow dragon*” due to the yellowing of shoots on citrus tree leaves, is a significant agricultural epidemic disease that causes serious economic losses for growers worldwide. Its presence has been reported in over 24 subtropical and tropical countries ranging from Asian nations, to Africa and the Americas (da Graça and Korsten 2004). According to a study from 2006 to 2010, HLB has had a significant negative economic impact on the Florida citrus industry, resulting in a loss of over 1.76 billion in economic activity traduced in 8, 200 jobs lost (Hodges and Spreen 2012). In recent years, orange production in Florida has descended from 242 million boxes in 2004, before the first report of HLB, to 68.9 million boxes in 2016 Gasparoto et al. (2018).

The disease is caused by a bacterium known as *Candidatus Liberibacter spp* which can be transmitted by the Asian Citrus Psyllid (ACP) named *Diaphorina citri* (Wang and Trivedi 2013). In the presence of the ACP vector, HLB can spread rapidly throughout a citrus grove, causing significant damage and greatly reducing crop yields. Effective control management of HLB disease includes several measures, such as inspecting and removing infected plants, using insecticide sprays to decrease ACP vector population, planting healthy nursery trees, among others (Gasparoto et al. 2018; Hall 2020).

Mathematical models, on the other hand, have demonstrated their efficacy as instruments for comprehending the intricate mechanisms entailed in both the propagation and management of HLB illness (Chiyaka et al. 2012; Gao et al. 2018; Zhang et al. 2020; Vilamiu et al. 2012). For instance, R. Taylor et al. proposed a compartment epidemic model in their study (Taylor et al. 2016) to understand the most cost-effective spraying strategy. The model includes compartments related to infectious trees, such as those that are asymptomatic or infected, and also tracks the count of roguing trees. Their findings suggest that increasing the number of days of spraying is the best strategy for thinning the orchard. Another compartment model proposed by Wang et al. (2014) includes logistic growth in ACP vector population and time-dependent parameters, simulating periodic environment in citrus orchards. The model considers susceptible and infected trees and vectors, making it a useful and straightforward method, but it does not consider the effect of other variables such as infectious asymptomatic trees. In another study, Zhang et al. (2021) proposed a detailed model for HLB transmission that includes all stages of the ACP vector life-span, resulting in a ten-dimensional nonlinear HLB system, which is computationally costly. Despite this, their analysis, based on optimal control, led the authors to conclude that a strategy based on insecticide and symptomatic trees removal is the most cost-effective strategy.

Further, final size estimation in compartmental epidemic models is an active research investigation area mainly focused on SIR epidemic models (Magal et al. 2018) or vector-borne disease SIR-SI models (Brauer 2017; Giménez-Mujica et al. 2023). The aim is to estimate a mathematical expression to assess the number of individuals who become infected with a disease during an epidemic outbreak (Andreasen 2011). For example, in Giménez-Mujica et al. (2022), the authors estimate the final size for the SIR epidemic model in a metapopulation network, and use this to design control strategies.

Since John Holland first introduced the idea in 1992 Holland (1992) as a way to obtain precise solutions to computationally challenging problems, the application of Genetic Algorithms (GA) has rapidly expanded to a broad range of practical problems. One of the benefits of using GAs is their ability to handle computationally challenging problems, particularly when the number of variables or parameters involved in an optimization problem significantly impacts its complexity. Genetic algorithms have been employed in epidemiological models to obtain the optimal parameter values that fit the data well, as demonstrated in Qiu et al. (2022). In a distinct approach, certain researchers have utilized meta-heuristic algorithms to estimate SEIR model parameters. For instance, particle swarm optimization was utilized for parameter estimation in the SIR epidemic model in Putra et al. (2019). Another study (Rojas-Delgado et al. 2021) employed the Firefly Algorithm, which is inspired by the social behavior of fireflies, for the same purpose.

In this paper, we propose a SAIR−SI compartment model for HLB transmission in a citrus orchard. The model accounts for roguing trees and utilizes a logistic growth approach for ACP vectors, as in Wang et al. (2014). By employing the next generation matrix, we derive a mathematical expression for the basic reproduction number $R_{0}$, and conduct a sensitivity analysis to identify the parameters that most influence the model. We then obtain an expression for the final size of the epidemic and develop a difference map equation that allows us to calculate the total number of roguing trees after the epidemic has subsided. This process is computationally inexpensive, and we use it in a genetic algorithm to determine the optimal combination of parameters that can effectively control HLB disease, as measured by the percentage of roguing trees. Furthermore, based on our analysis of the final size, we propose a risk measure for HLB transmission in the orchard and investigate the impact of control parameters on this measure.

## Preliminaries

The aim in this section is to describe the proposed mathematical model for HLB transmission in a given citrus orchard, named the SAIR−SI compartmental model. With this model, we will determine the local basic reproduction number by applying the Next Generation Matrix (NGM) approach.

### SAIR-SI compartmental model

Consider an isolated citrus orchard inhabited by a homogeneously mixing population of $N_{\tau }$ citrus trees and $N_{v}$ psyllid vectors. Specifically, we assume that the ACP *Diaphorina citri Kuwayama* is the primary transmitter of HLB within the orchard.

**Fig. 1:**
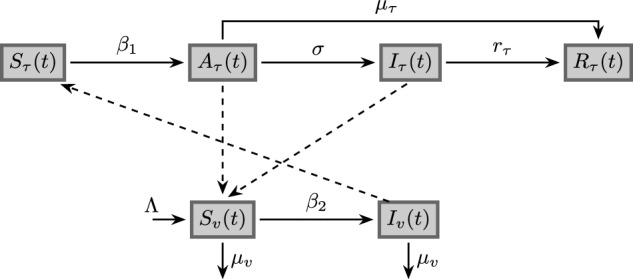
Compartmental diagram of the SAIR−SI model (Eqs. 1–2)

To model the spread of HLB within the orchard, we use a Susceptible-Asymptomatic-Infected-Roguing (SAIR) compartmental model that divides the citrus trees population into four categories at any time instant *t*: $S_{\tau }(t)$ susceptible trees, $A_{\tau }(t)$ infectious and asymptomatic trees, $I_{\tau }(t)$ infectious and symptomatic trees, and $R_{\tau }(t)$, represents the practice of roguing trees, which in the agricultural context refers to identifying and removing infected trees.

On the other hand, HLB is spread exclusively when ACP vectors feed on the phloem of the trees, and once infected, the psyllids maintain the infection for their entire lifetime. In this context, we divide the ACP vectors state into two groups: susceptible $S_{v}(t)$ and infectious $I_{v}(t)$. We assume that the total population of ACP vector within the orchard $N_{v}(t) = S_{v}(t) + I_{v}(t)$ grows logistically, with a carrying capacity given by the total number of citrus trees already present in the orchard multiplied by the abundance of psyllids per tree *m* Wang et al. (2014).

Hence, we employ a SAIR model for citrus trees and a SI (Susceptible-Infectious) model for the ACP vectors, which are described by the following set of nonlinear differential equations and represented in the compartmental diagram shown in Fig. 1:

1 $$\begin{aligned} &  \text {Trees} \left\{ \begin{array}{lll} \dfrac{d {S}_{\tau }}{dt} & =& - \beta _{1}\dfrac{S_{\tau }\cdot I_{v}}{N_{\tau }} \,, \\ \\ \dfrac{d A_{\tau }}{dt} & =& \beta _{1}\dfrac{S_{\tau }\cdot I_{v}}{N_{\tau }} - (\sigma + \mu _{\tau })A_{\tau } \,, \\ \\ \dfrac{d I_{\tau }}{dt} & =& \sigma A_{\tau } - r_{\tau } I_{\tau } \,, \\ \\ \dfrac{d R_{\tau }}{dt} & =& \mu _{\tau }A_{\tau } + r_{\tau }I_{\tau } \,; \end{array} \right. \end{aligned}$$

2 $$\begin{aligned} &  \text {ACP} \left\{ \begin{array}{lll} \dfrac{d {S}_{v} }{dt} & =& \Lambda (N_{v}) - \beta _{2} \dfrac{S_{v}\cdot (A_{\tau } + I_{\tau })}{N_{\tau }} - \mu _{v}S_{v} \,, \\ \\ \dfrac{d {I}_{v} }{dt} & =& \beta _{2} \dfrac{S_{v}\cdot (A_{\tau } + I_{\tau })}{N_{\tau }} - \mu _{v}I_{v} \,; \\ \end{array} \right. \end{aligned}$$

where $\mu _{\tau }$ denotes the average life expectancy of citrus trees, which typically falls within the range of 20 to 30 years according to Zhang et al. (2020). Similarly, the parameter $\mu _{v}$ represents the natural mortality rate of ACP vectors. The progression rate from asymptomatic to symptomatic infection in trees is represented by the parameter $\sigma$, while the transmission rate of HLB from infected ACP vector to susceptible trees is given by $\beta _{1}$, and the transmission rate of HLB from infected trees to ACP vectors is given by $\beta _{2}$. It should be noted that the SAIR-SI model includes the parameter $r_{\tau }$, which represents the degree of effectiveness of human vigilance, which entails closely monitoring the orchard to detect and replace citrus trees that exhibit symptoms of HLB, such as yellowing or chlorotic blotches on leaves, or misshapen fruits.

It is important to emphasize that our SAIR-SI model operates on a monthly time scale, a crucial choice that significantly impacts how we address the reintroduction of rogued trees into the orchard. This choice comes with a key consideration: we assume that, upon removing an infected tree, the grower replaces it with a new, young, and susceptible tree. This practice ensures that the growers maintain a constant total number of citrus trees, denoted as $N^{*}_{\tau }$, within the orchard. However, we also assume that these newly introduced trees require a certain amount of time to mature and become productive. This assumption is in line with typical agricultural practices, where the immediate replacement of infectious symptomatic trees with susceptible and productive ones is infrequent, especially when dealing with monthly intervals.

Furthermore, it’s important to note that our method of counting roguing trees in compartment $R_{\tau }$ does not include the newly introduced young trees. Then, when we sum all the equations for the citrus tree compartments (Eq. 1), we find that the derivative of $N_{\tau }$ is zero. This signifies that the total number of trees, $N_{\tau }$, remains constant at all times, including the trees that have been removed. It’s important not to confuse this with $N^{*}_{\tau }$, which includes the total number of trees into the orchard, including the new trees that replace the infected ones.

On the other hand, the rate at which new monthly-born ACPs are added to the orchards is determined by the logistic growth equation:

$$\begin{aligned} \Lambda (N_{v}) = \alpha (S_{v} + I_{v}) \cdot \Big ( 1 - \dfrac{S_{v} + I_{v}}{ m \cdot N^{*}_{\tau }} \Big ) \,. \end{aligned}$$

Here, we determine the orchard’s carrying capacity by taking the product of two factors: the maximum abundance of ACP vectors per citrus tree, denoted as *m*, and $N^{*}_{\tau }$ which represents the number of citrus trees already present in the orchard. We assume that this number remains constant and corresponds to the total number of citrus trees managed by the growers, encompassing the young citrus trees replacing the symptomatic ones. Notably, young citrus trees are most susceptible to HLB infections, since psyllids require young, actively-growing foliage (flush) for development, and their populations reach their highest levels during flush periods (Bové 2006).

**Table 1:** Description of the parameters of the SAIR-SI model (Eqs. 1–2), along with the corresponding numerical values reported in references for an orchard without control intervention

Parameter	Description	Baseline value	Range	References
$N^{*}_{\tau }$	Total number of citrus trees already present in the orchard	2000	1000–2000	Assumed
$\mu _{\tau }$	Natural mortality rate of citrus trees ($month^{-1}$)	0.0033	0.00275–0.00417	Wang et al. (2014); Zhang et al. (2020)
$\mu _{v}$	Natural mortality rate of ACP ($month^{-1}$)	0.5	0.5–1	Wang et al. (2014); Zhang et al. (2020); Pérez-Artiles et al. (2017)
$\beta _{1}$	HLB transmission rate from infected ACP vector to susceptible tree (feeding rate of vector times prob. of transmission)	0.1	0.5–1	Taylor et al. (2016); d’A Vilamiu et al. (2013)
$\beta _{2}$	HLB transmission rate from infected tree to susceptible ACP vector (feeding rate of vector times prob. of transmission)	0.365	0.02–0.65	Taylor et al. (2016); d’A Vilamiu et al. (2013)
$r_{\tau }$	Effectiveness of orchard vigilance	0.7	0.1–0.9	Assumed
$\sigma$	Rate at which asymptomatic infected citrus tree becomes symptomatic ($month^{-1}$)	0.2	0.155–0.99	Chiyaka et al. (2012); Zhang et al. (2020)
$\alpha$	Intrinsic growth rate of the D. citri population (*month*)	2.25	120–1000	Beloti et al. (2013); Sule et al. (2012)
*m*	Maximum abundance of ACP vectors per citrus tree	400	120-1000	Wang et al. (2014)

In Table 1, the description of the parameters of the SAIR-SI model (Eqs. (1–2) is summarized, along with the corresponding numerical values reported in references. It is noteworthy that the transmission rate of HLB from infected ACP vectors to susceptible citrus trees is determined by the product of the feeding rate of ACP vectors, denoted by *a*, which is estimated as $0.05*365/12$ according to Taylor et al. (2016). However, as noted by Vilamiu et al. (2013) in d’A Vilamiu et al. (2013), there is a lack of agreement on the appropriate value for this parameter; even that, the available literature suggests that this probability is exceedingly low.

**Fig. 2:**
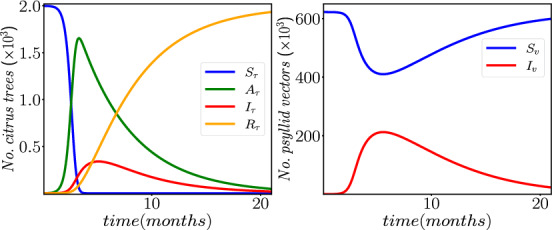
Numerical solution to the SAIR−SI model (Eqs.1–2) using the fourth order Runge–Kutta method (RK4) and the baseline parameter values provided in Table 1

To illustrate the dynamics of HLB in an isolated orchard, we present, in Fig. 2, the numerical solution to the SAIR−SI model using the fourth-order Runge–Kutta method (RK4) and the baseline parameter values in Table 1. The initial conditions are set as $S_{\tau }(t=0)=N_{\tau }$, $A_{\tau }(t=0)=1$, $I_{\tau }(t=0)=0$, $R_{\tau }(t=0)=0$, $S_{v}(t=0)= N_{v}^{0}$, and $I_{v}(t=0)=0$, where $N_{v}^{0} = |1 - \frac{\mu _{v}}{\alpha }|\cdot m \cdot N_{\tau }$ represents the number of susceptible ACP vectors in the orchard at the beginning of HLB disease, which we assume starts at the equilibrium point of ACP population. One should note that in order to ensure a biological sense, the absolute value of $1 - \mu _{v}/\alpha$ is taken to guarantee a positive population at the onset of the disease.

Based on the results of the numerical simulation, it can be observed that the maximum percentage of asymptomatic infectious trees reaches 82.71 % during the first third or fourth months after the introduction of the first infected tree into the orchard. However, the maximum percentage of symptomatic infectious trees is observed to reach 17% of the total population at around five months.

Furthermore, it is worth mentioning that the overall number of roguing trees steadily approaches the total number of citrus trees in the orchard, denoted as $N_{\tau }$. This implies that complete eradication of HLB transmission necessitates the removal of 100% of the citrus trees, which is not a viable solution in practical scenarios.

In summary, the numerical simulation results demonstrate the dynamics of HLB disease transmission in the citrus orchard and highlight the importance of timely intervention measures to minimize its impact on the crop.

## Basic reproduction number and parametric sensitivity analysis

### Basic reproduction number

The basic reproduction number $R_{0}$ is a crucial indicator that describes the progression of an epidemic. It predicts the number of secondary infections that a single infected citrus tree can cause in a completely susceptible population. A higher $R_{0}$ suggests a greater likelihood of rapid disease spread through a population. When $R_{0} < 1$, the number of infected citrus trees decreases, indicating a decrease in the severity of the disease, whereas, above this threshold, the disease proliferates in the orchard (Martcheva 2015).

The NGM method is a technique used to calculate a mathematical expression for $R_{0}$ by treating the infection process as a demographic process where newly infected individuals are added or removed (Diekmann et al. 2009). We employ this methodology to evaluate the $R_{0}$ for the SAIR−SI model. Subsequently, we perform a global parametric sensitivity analysis of both $R_{0}$ and SAIR−SI model using the Latin Hypercube Sampling (LHS) and partial rank correlation coefficients (PRCCs) techniques.

The initial step in the NGM method involves dividing the compartmental model into two sub-systems: the infected sub-system, also referred to as the infected compartments, and the disease-free sub-system. For the SAIR−SI model, the infected sub-system comprises the state variables $x = (x_{1},x_{2},x_{3}) = (A_{\tau }, I_{\tau },I_{v}) \in {\mathbf{R}}^{3}$, while the state variables $y = (S_{\tau }, R_{\tau },S_{v}) \in {\mathbf{R}}^{3}$ make up the disease-free sub-system. The right-hand side of the infected sub-system is then separated as follows:

$$\begin{aligned} \begin{array}{lll} \dfrac{d A_{\tau }}{dt} & =& \underbrace{\beta _{1}\dfrac{S_{\tau }\cdot I_{v}}{N_{\tau }}}_{\mathbf{F}_{1}} \quad \underbrace{ - (\sigma + \mu _{\tau })A_{\tau } }_{ \mathbf{V}_{1}} \,, \\ \\ \dfrac{d I_{\tau }}{dt} & =& \underbrace{\sigma A_{\tau } - r_{\tau } I_{\tau }}_{\mathbf{V}_{2}} \,, \\ \\ \dfrac{d {I}_{v} }{dt} & =& \underbrace{\beta _{2} \dfrac{S_{v}\cdot (A_{\tau } + I_{\tau })}{N_{\tau }}}_{\mathbf{F}_{3}} \underbrace{- \mu _{v}I_{v}}_{\mathbf{V}_{3}} \,; \\ \end{array} \end{aligned}$$

where ${\mathbf{F}}_{i}$ (for $i=1,2,3$) are the transmission terms and ${\mathbf{V}}_{i}$ the births, deaths, disease progression or recovery terms.

Next, the NGM methodology involves constructing the following matrices:

$$\begin{aligned} \begin{array}{lll} \mathcal {F} & =& \displaystyle \left. \left[ {\frac{\partial {\mathbf{F}}_{i}}{\partial x_{k}}} \right| _{\tiny {P}} \right] _{3 \times 3} = \begin{pmatrix} 0 &\quad 0 &\quad \beta _{1} \\ 0 &\quad 0 &\quad 0 \\ \beta _{2}\dfrac{N^{0}_{v}}{N_{\tau }} &\quad \beta _{2}\dfrac{N^{0}_{v}}{N_{\tau }} &\quad 0 \end{pmatrix}; \\ \\ \mathcal {V} & =& \left. \left[ {\frac{\partial {\mathbf{V}}_{j}}{\partial x_{k}}} \right| _{\tiny {P}} \right] _{3 \times 3} = \begin{pmatrix} -\sigma -\mu _{\tau } &\quad 0 &\quad 0 \\ \sigma &\quad -r_{\tau } &\quad 0 \\ 0 &\quad 0 &\quad -\mu _{v} \end{pmatrix} \,; \end{array} \end{aligned}$$

with $x_{k} = (A_{\tau }, I_{\tau }, I_{v}) \in R^{3}$ and $P = (N_{\tau }, 0, 0, 0, N^{0}_{v}, 0 ) \in {\mathbf{R}}^{6}$ represents the disease-free equilibrium state (*DFE*).

The matrix $K = -\mathcal {F}\mathcal {V}^{-1}$ is the NGM (Diekmann et al. 2009) which, for the SAIR−SI model, becomes:

$$\begin{aligned} K = \begin{pmatrix} 0 &\quad 0 &\quad \dfrac{\beta {1}}{\mu _{v}} \\ 0 &\quad 0 &\quad 0 \\ \dfrac{ \beta _{2} N_{v}^{0} (r_{\tau } + \sigma )}{ r_{\tau } N_{\tau } (\sigma + \mu _{\tau })} &\quad \beta _{2}\dfrac{N_{v}^{0}}{r_{\tau }N_{\tau }} &\quad 0 \end{pmatrix}. \end{aligned}$$

Therefore, the basic reproduction number is represented by the spectral radius $\rho (K)$ of the NGM. To obtain the mathematical expression of $R_{0}$, we solve the characteristic polynomial $p(\lambda ) = det( K - \lambda \mathbb {I}_{3}) = 0$, where $\mathbb {I}_{3}$ is the $3 \times 3$ identity matrix. If we take the initial number of ACP vectors in the orchard as $N_{v}^{0} = |1 - \mu _{v}/\alpha |\cdot m \cdot N_{\tau }$ (at equilibrium), then we obtain:

3 $$\begin{aligned} \begin{array}{llllll} R_{0} & = & \rho (K) & = & \sqrt{\dfrac{\beta _{1}}{\sigma + \mu _{\tau }} \cdot \dfrac{\beta _{2}}{\mu _{v}} \cdot \dfrac{N^{0}_{v}}{N_{\tau }} \cdot \Big ( \dfrac{\sigma }{r_{\tau }} + 1\Big )} \,,\\ \\ & & & = & \sqrt{R_{\tau v} \cdot R_{v \tau } \cdot m \cdot \Big | 1 - \dfrac{\mu _v}{\alpha }\Big | \cdot \Big ( \dfrac{\sigma }{r_{\tau }} + 1\Big )} \,; \end{array} \end{aligned}$$

where $R_{\tau v} = \beta _{1}/(\sigma + \mu _{\tau })$ denotes the ratio of new infections generated by a single asymptomatic infectious tree during its stay in the orchard, which is given by $1/(\sigma + \mu _{\tau })$; while $R_{v\tau } = \beta _{2}/\mu _{v}$ is the ratio of new infectious caused by an ACP vector during its lifespan, which is given by $1/\mu _{v}$. Based on the baseline parameter values provided in Table 1, the basic reproduction number of the orchard is $R_{0} \approx 12$, which is greater than 1. This explains the cause of the HLB disease observed in the example depicted in Fig. 2.

### Sensitivity and uncertainty analysis

An important feature of the SAIR-SI epidemic model for HLB transmission in an orchard is that its dynamics is influenced by the combination of values of its parameters. To assess the impact of this uncertainty and sensitivity of model outcomes to parameter variations, we perform a global sensitivity analysis using Latin Hypercube Sampling (LHS) and partial rank correlation coefficients (PRCCs) methodologies.

**Fig. 3:**
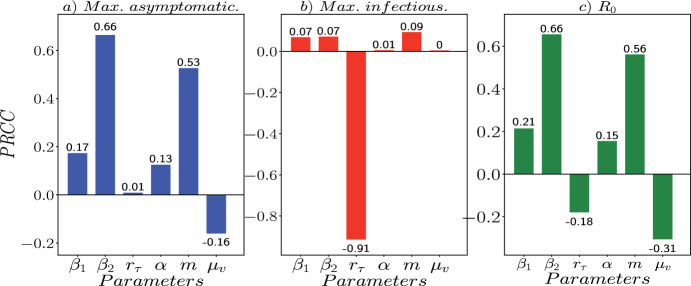
Parametric sensitivity analysis with partial rank correlation coefficients (PRCCs) for the SAIR-SI epidemic model

Latin Hypercube Sampling (LHS) is a stratified sampling technique that allows for an efficient and thorough exploration of parameter space. This sampling technique ensures that each parameter value is sampled equally across its range, reducing the likelihood of missing important parameter combinations.

**Fig. 4:**
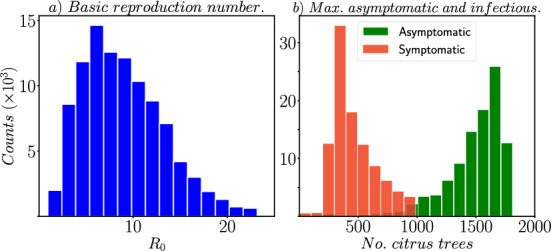
Histograms obtained from Latin hypercube sampling (LHS) using a sample size of 100,000 for parameter values. **a** shows the histogram of $R_{0}$ (Eq. 3); **b** displays the histograms for the maximum number of asymptomatic (in red) and symptomatic (in green) cases observed for each parameter sampling

Furthermore, PRCCs are useful in identifying which parameters have the strongest influence on the model output, and how changes in each parameter affect the model outcome. With the PRCCs method, we can measure the strength of the linear relationship between the SAIR−SI model output and each input parameter while controlling for the effects of other parameters. A PRCC value of zero indicates that there is no linear correlation between the model output and the input parameter, while a value of -1 or 1 indicates a strong negative or positive correlation, respectively.

To generate the LHS matrices, we focused on six of the nine parameters outlined in Table 1: $\beta _{1}$, $\beta _{2}$, $r_{\tau }$, $\alpha$, $\mu _{v}$, and *m*. In this work we consider them as control parameters. To obtain uniformly distributed values for these parameters according to the LHS method, we generated $10^{5}$ samples using the *pyDOE* Python library (Baudin 2021). For each sample (i.e., combination of parameter values), we perform a numerical simulation of the SAIR-SI model and recorded the resulting outputs, which included the peak of the curves of infectious compartments (which we call the maximum number of asymptomatic and symptomatic infectious trees) and the numerical value of $R_{0}$ (Eq. 3).

To perform sensitivity and uncertainty analyses, we computed the partial rank correlation coefficients (PRCCs) of the model outputs using the LHS method. As depicted in Fig. 3a, we observe that the maximum number of asymptomatic citrus trees exhibits a negative correlation with the parameter $\mu _{v}$, implying that an increase in this parameter reduces the maximum number of asymptomatic trees. Moreover, the parameter $r_{\tau }$ demonstrates a correlation close to zero, suggesting that variations in orchard vigilance have a negligible impact on the maximum number of asymptomatic trees compared to other parameters, with $\beta _{2}$ exhibiting the highest positive correlation.

In Fig. 3b, we note a negative correlation with the effectiveness of orchard vigilance $r_{\tau }$, indicating that a decrease in vigilance leads to an increase in the number of symptomatic infectious citrus trees. Additionally, in Fig. 3c, due to their negative correlation, increasing $r_{\tau }$ and $\mu _{v}$ can decrease the value of $R_{0}$, making these parameters important candidates for control measures. Furthermore, among the parameters with positive correlation, we observe that the most sensitive parameters showing the highest correlations are the parameter $\beta _{2}$ and *m*, both related to the control of ACP vectors.

In Fig. 4a, it is observed that for approximately $0.01 \%$ of the parameter distribution, $R_{0}<1$, indicating the potential occurrence of persistent HLB bacterial infection. Moreover, based on calculations, the mean and standard deviation (std) of $R_{0}$ are 9 and 4.36, respectively. By analyzing the $10^{5}$ combinations of parameters, it was found that the parameter values that produce an output where $R_{0}<1$ have an average value of $\beta _{1} = 0.0578$, $\beta _{2} = 0.0368$, $r_{\tau } = 0.655$, $\alpha = 1.731$, $m = 126.73$, and $\mu _{v} = 0.911$. Notably, parameters $\beta _{1}$ $\beta _{2}$ should be approximately $10^{-2}$ to ensure effective mitigation of HLB in the orchard; and the natural mortality rate of ACP ($\mu _{v}$) is approximately one month, as reported in Pérez-Artiles et al. (2017).

**Fig. 5:**
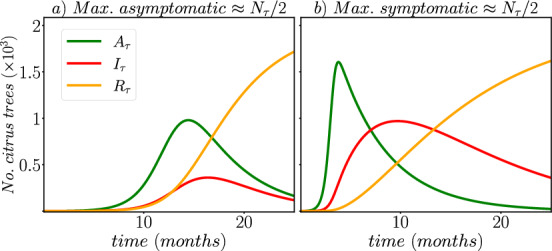
**a** The peak of the asymptomatic curve ($A_{\tau }(t)$) is close to ($N_{\tau }/2$), from which the average value of the corresponding parameters are: $\beta _{1} = 0.0676$, $\beta _{2} = 0.0687$, $r_{\tau } = 0.464$, $\alpha = 2.185$, $m = 356$, and $\mu _{v} = 0.817$. **b** The peak of the symptomatic curve ($I_{\tau }(t)$) is close to ($N_{\tau }/2$), from which the average value of the corresponding parameters are: $\beta _{1} = 0.079$, $\beta _{2} = 0.332$, $r_{\tau } = 0.104$, $\alpha = 2.23$, $m = 535$, and $\mu _{v} = 0.776$

In Fig. 4b we observe the distribution of the maximum number (the peak of the curve) for the asymptomatic and symptomatic infectious compartments. The mean and standard deviation for the asymptomatic compartment are 1484 and 259, respectively. Similarly, for the symptomatic compartment, the mean and standard deviation values are 465 and 182, respectively. We find that, from the $10^{5}$ combinations of parameters, $5\%$ corresponds to values from which the peak of the asymptomatic curve is less than half the number of citrus tress ($< N_{\tau }/2$), from which the average value of the corresponding parameters are: $\beta _{1} = 0.0676$, $\beta _{2} = 0.0687$, $r_{\tau } = 0.464$, $\alpha = 2.185$, $m = 356$, and $\mu _{v} = 0.817$. Similarly, $0.86\%$ of the $10^{5}$ samples, we get a scenario where the maximum number of symptomatic infectious citrus trees exceeds the half population ($> N_{\tau }/2$). In this case, the average values of the corresponding parameters are: $\beta _{1} = 0.079$, $\beta _{2} = 0.332$, $r_{\tau } = 0.104$, $\alpha = 2.23$, $m = 535$, and $\mu _{v} = 0.776$. It is worth noting that in this scenario, the effectiveness of orchard vigilance ($r_{\tau }$) is low. Figure 5 illustrates the dynamics of the HLB disease in both scenarios.

## The final size of the HLB disease

In the context of mathematical epidemiology, the final size equation provides valuable information regarding the overall percentage of susceptible hosts that were infected during an epidemic. Essentially, it can be viewed as an indicator of the severity of the epidemic, as it measures the changes in the size of the infectious group relative to the size of the susceptible group throughout the duration of the outbreak (Andreasen 2011). In this section, we derive, in terms of $R_{0}$, a mathematical expression for the final size equation for the SAIR-SI model (Eqs. 1–2), which enables us to measure the severity of the HLB disease in an orchard. Additionally, we include an expression that quantifies the amount of risk during the disease. This measure can provide growers with valuable information to make informed decisions and take better control measures.

### Final size for infected ACP vectors and risk

The aim of this section is to obtain an expression for the integral of compartment $I_{v}(t)$ from zero to infinity, based on Eqs. (1–2). This integral represents the cumulative fraction of ACP vectors that have been infected by HLB bacteria. By expressing this integral in terms of $R_0$, we can evaluate the potential risk for continued transmission in both our own orchard and neighboring orchards.

Since $S_{v}(t) = N_{v} - I_{v}(t)$, Eq. (2) for ACP vectors can be written as the following single equation:

$$\begin{aligned} \dfrac{dI_{v}}{dt} = \beta _{2}(N_{v} - I_{v})\dfrac{A_{\tau } + I_{\tau }}{N_{\tau }} - \mu _{v}I_{v} ; \end{aligned}$$

solving for $A_{\tau } + I_{\tau }$ we get (Giménez-Mujica et al. 2022, 2023):

$$\begin{aligned} A_{\tau } + I_{\tau } = \dfrac{N_{\tau }}{\beta _{2}N_{v}(1-I_{v}/N_{v})}\cdot \dfrac{dI_{v}}{dt} + \dfrac{N_{\tau }\mu _{v}}{\beta _{2}N_{v}(1-I_{v}/N_{v})}\cdot I_{v} \end{aligned}$$

$$\begin{aligned}  \approx \dfrac{N_{\tau }}{\beta _{2}N_{v}}\cdot \dfrac{dI_{v}}{dt} + \dfrac{N_{\tau }\mu _{v}}{\beta _{2}N_{v}}\cdot I_{v} ; \end{aligned}$$

where, by considering that $I_{v}/N_{v} < 1$, we approximate the term $1/(1-I_{v}/N_{v})$ by a series expansion and ignore higher order terms. Then, integrating both sides and considering that $I_{v}(t)$ converges to zero when $t \rightarrow \infty$, we obtain:

4 $$\begin{aligned} \int _0^\infty I_{v}(t)dt = \dfrac{\beta _{2}N_{v}}{N_{\tau }\mu _{v}} \int _0^\infty (A_{\tau } + I_{\tau })dt \,, \end{aligned}$$

where we have assumed that $I_{v}(0) = 0$, i.e., at the beginning of the disease there are no symptomatic infectious ACP vectors. Our objective is to determine both integrals on the right-hand side of Eq. (4).

First, note that from the equation for the roguing compartment $R_{\tau }$ in Eq. (2), we get, after integrating both sides from 0 to infinity and considering that its initial condition is $R_{\tau }(0) = 0$ (indicating that no trees were removed at the beginning of the disease):

$$\begin{aligned} R_{\tau }(\infty ) = \mu _{\tau } \int _0^\infty A_{\tau }(t)dt + r_{\tau } \int _0^\infty I_{\tau }(t) dt \,. \end{aligned}$$

which implies:

5 $$\begin{aligned} \int _0^\infty I_{\tau }(t) dt = \dfrac{R_{\tau }(\infty )}{r_{\tau }} - \dfrac{\mu _{\tau }}{r_{\tau }}\int _0^\infty A_{\tau }(t)dt \,. \end{aligned}$$

By summing the compartments $I_{\tau }$ and $R_{\tau }$ of Eq. (1), we get:

$$\begin{aligned} \dfrac{dR_{\tau }}{dt} + \dfrac{dI_{\tau }}{dt} = (\sigma + \mu _{\tau })A_{\tau }; \end{aligned}$$

from which, after integrating both sides and considering $R_{\tau }(0) = 0$:

6 $$\begin{aligned} \int _0^\infty A_{\tau }(t) dt = \dfrac{R_{\tau }(\infty )}{\sigma +\mu _{\tau }} - \dfrac{I_{\tau }(0)}{\sigma +\mu _{\tau }} \,. \end{aligned}$$

Substituting Eqs. (5) and (6) in Eq. (4) we get

$$\begin{aligned} \int _0^\infty I_{v}(t) dt&\approx \dfrac{\beta _{2}N_{v}}{\mu _{v}N_{\tau }}\cdot \dfrac{R_{\tau }(\infty )}{r_{\tau }} + \dfrac{\beta _{2}N_{v}}{\mu _{v}N_{\tau }}\cdot \dfrac{r_{\tau } - \mu _{\tau }}{r_{\tau }} \int _0^\infty A_{\tau }(t) dt \\&= \dfrac{\beta _{2}N_{v}}{\mu _{v}N_{\tau }}\cdot \dfrac{R_{\tau }(\infty )}{r_{\tau }} \left ( 1 + \dfrac{r_{\tau } - \mu _{\tau }}{\sigma + \mu _{\tau }}\right ) + \dfrac{\mu _{\tau } - r_{\tau }}{\mu _{\tau } + \sigma } \cdot \dfrac{\beta _{2}N_{v}}{r_{\tau }\mu _{v}N_{\tau }}\cdot I_{\tau }(0) \\&= \dfrac{\beta _{2}N_{v}}{\mu _{v}N_{\tau } (\sigma +\mu _{\tau })}\cdot \left( \dfrac{\sigma }{r_{\tau }} + 1 \right )\cdot R_{\tau }(\infty ) + \dfrac{\mu _{\tau } - r_{\tau }}{\mu _{\tau } + \sigma } \cdot \dfrac{\beta _{2}N_{v}}{r_{\tau }\mu _{v}N_{\tau }} \cdot I_{\tau }(0) \,. \end{aligned}$$

Then, using the $R_{0}$ given in Eq. (3), we get the following expression of the integral of $I_{v}(t)$, that is, from the cumulative fraction of ACP vectors that have been infected:

7 $$\begin{aligned} \int _0^\infty I_{v}(t) dt \approx \dfrac{R^{2}_{0}}{\beta _{1}}\cdot \left ( R_{\tau }(\infty ) + \dfrac{\mu _{\tau }-r_{\tau }}{\sigma +r_{\tau }}\cdot I_{\tau }(0) \right ) . \end{aligned}$$

It is worth remarking the dependence of the above integral with the basic reproduction number $R_{0}$ and $R_{\tau }(\infty )$, which represent the total number of citrus trees that have been infected. The term $R_{\tau }(\infty )$ is what is usually called, in mathematical epidemiology, the final size of the disease transmission. In the next section, we analyze this term and derive an expression to estimate its value.

Given the expression (7), we define the risk in an orchard as the likelihood of further transmission of HLB bacteria. The higher the cumulative fraction of infected ACP vectors per citrus tree in an orchard, the greater the risk of the disease spreading rapidly within the orchard and potentially to neighboring orchards. Therefore, a high cumulative fraction of infected ACP vectors signifies a greater need for appropriate control measures to be implemented to prevent further spread and reduce the risk of extensive damage to the orchard.

In this context, we define the risk in a citrus orchard as:

8 $$\begin{aligned} Risk = \dfrac{1}{N_{\tau }} \int _0^\infty I_{v}(t) dt \approx \dfrac{R^{2}_{0}}{N_{\tau } \beta _{1}}\cdot \left ( R_{\tau }(\infty ) + \dfrac{\mu _{\tau }-r_{\tau }}{\sigma +r_{\tau }}\cdot I_{\tau }(0) \right ) . \end{aligned}$$

Since $R_{\tau }(\infty ) \le N_{\tau }$ and $I_{\tau }(t) \approx 0$ as $t \rightarrow \infty$, we could observe that the risk is bounded by the $R^{2}_{0}/\beta _{1}$ value.

**Fig. 6:**
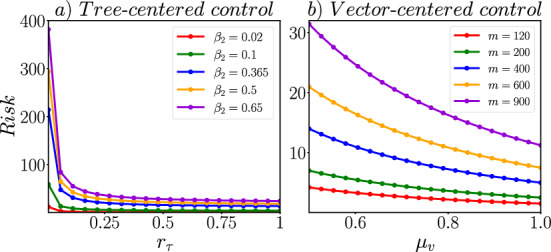
Risk value (Eq. 8) under two different control strategies: **a** tree-centered and **b** vector-centered

Through $R_{0}$ sensitivity analysis (Fig. 3), we have identified four potential control parameters: $\beta _{2}$, $r_{\tau }$, *m*, and $\mu _{v}$. The first two parameters focus on reducing the contact between citrus trees and ACP vectors. The latter two parameters aim to reduce the population of ACP vectors through the use of chemical substances. We refer to the first set of control strategies, which utilize only $\beta _{2}$ and $r_{\tau }$, as the tree-centered strategy. The second set of control strategies, which target ACP vectors and involves the parameters *m* and $\mu _{v}$, is referred to as the vector-centered strategy.

In Fig. 6, we demonstrate the numerical value of the risk expression (8) by varying the corresponding parameters in a control strategy that is either tree-centered or vector-centered. To determine the value of $R_{\tau }(\infty )$ in the risk expression, we numerically solve the SAIR-SI model with the parameters fixed at their baseline values, as given in Table 1, and extract the last value of the variable $R_{\tau }$ from the time series as the final size. It is worth noting, as shown in Fig. 6, the risk is reduced in both control strategies when more orchard vigilance is implemented (i.e., when $r_{\tau }$ is increased in a tree-centered control strategy) or when the mortality rate of ACP increases due to a fumigation plan (i.e., when $\mu _{v}$ is increased in a vector-centered control strategy).

### Final size for infected citrus trees

In this section, we obtain a mathematical expression for the final size of an orchard, denoted by $R_{\tau }(\infty )$, using Eq. (7). The final size represents the total number of citrus trees that become infected with HLB bacteria during an epidemic in a specific orchard. Our goal is to develop a numerical method based on difference equations to estimate this term.

Consider the equation for the compartment $S_{\tau }$ in Eq. (1). It could be rewritten as

$$\begin{aligned} \int _0^\infty \dfrac{dS_{\tau }}{S_{\tau }} = -\dfrac{\beta _{1}}{N_{\tau }}\int _0^\infty I_{v}(t)dt; \end{aligned}$$

using Eq. (7), we get:

$$\begin{aligned} ln\left ( \dfrac{S_{\tau }(\infty )}{S_{\tau }(0)}\right ) \approx -\dfrac{R^{2}_{0}}{N_{\tau }}\cdot \left ( R_{\tau }(\infty ) + \dfrac{\mu _{\tau }-r_{\tau }}{\sigma +r_{\tau }}\cdot I_{\tau }(0) \right ); \end{aligned}$$

where $S_{\tau }(\infty ) = \lim _{t \rightarrow \infty } S_{\tau }(t)$ and $S_{\tau }(0)$ is the total number of susceptible citrus trees at the beginning of the HLB epidemic. But $N_{\tau } = S_{\tau }(\infty ) + A_{\tau }(\infty ) + I_{\tau }(\infty ) + R_{\tau }(\infty )$ since the number of citrus trees remains constant all the time. Then, considering that $\lim _{t \rightarrow \infty } A_{\tau }(t) \approx 0 \approx \lim _{t \rightarrow \infty } I_{\tau }(t)$, we obtain $S_{\tau }(\infty ) = N_{\tau } - R_{\tau }(\infty )$. Thus, we obtain the following mathematical expression of the HLB epidemic final size in an orchard:

9 $$\begin{aligned} R_{\tau }(\infty ) \approx N_{\tau } - S_{\tau }(0)\exp \left( -\dfrac{R^{2}_{0}}{N_{\tau }}\cdot \left ( R_{\tau }(\infty ) + \dfrac{\mu _{\tau }-r_{\tau }}{\sigma +r_{\tau }}\cdot I_{\tau }(0) \right ) \right); \end{aligned}$$

It should be noted that while Eq. (9) provides an explicit form for the final size of the HLB epidemic, finding an analytical solution to it is a difficult problem due to its implicit dependence on the term $R_{\tau }(\infty )$. To address this, two complementary approaches can be considered. First, following classical fixed-point theory, we can reformulate Eq. (9) as an iterative difference equation, exploiting the fact that $R_{\tau }(\infty )$ converges under certain conditions (Giménez-Mujica et al. 2022, 2023). This allows us to compute the solution numerically and provides a robust framework for exploring properties such as existence, uniqueness, and convergence, offering deeper theoretical insights into the behavior of the system. This method is particularly advantageous for extending the analysis to higher-dimensional or more complex epidemic models, as fixed-point theorems ensure the generalization of the approach.

However, recognizing the potential for a faster and more direct solution, we have also explored the analytical resolution of Eq. (9) using the Lambert W function. By introducing a change of variable, the equation can be solved explicitly, yielding an expression for $R_{\tau }(\infty )$ in closed form. This approach simplifies the computational aspect significantly and is detailed in Appendix A, where the full derivation is provided.

Throughout this work, we emphasize the fixed-point approach, as it offers a richer theoretical framework for future generalizations, while still acknowledging the efficiency and utility of the Lambert W function for rapid computation. In this sense, let $z^{k}$ be the number of roguing citrus trees and $k = 0,1,\ldots q$, with $q \in \mathcal {N}$, being an index that represents the iteration number. We propose the following difference equation:

10 $$\begin{aligned} z^{k+1} = N_{\tau } - S_{\tau }(0)e^{-\gamma \cdot (z^{k} + \delta )}; \end{aligned}$$

where $\gamma = R^{2}_{0}/N_{\tau }$, $\delta = (\mu _{\tau } - r_{\tau })/(\sigma + r_{\tau })$ and we are setting $I(0) = 1$. With this equation, we explore numerically the parameter conditions under which we could arrive to the half or another percentage of roguing trees as the final size of the HLB disease.

## Control strategies based on genetic algorithm

In this section, we present a methodology to identify the optimal combination of control parameters for roguing the least number of citrus trees in an HLB disease. Our proposed methodology aims to minimize the number of trees to be rogued at the end of the HLB disease. Specifically, the optimization problem to be addressed is:

**Problem**: Let the desired final epidemic size be denoted by $z^{\text {obj}}_{\tau } = \phi N_{\tau }$, where $\phi \in [0, 1]$ represents the target percentage of trees to be rogued at the end of the HLB epidemic. Determine the combination of control parameters $p_{1}$ and $p_{2}$ such that

$$\begin{aligned} \min \limits _{p_{1}, p_{2}} | R_{\tau }(\infty ) - z^{obj}_{\tau }| \,; \end{aligned}$$

subject to the SAIR−SI model (Eqs.1–2). Here, the parameters $p_1$ and $p_2$ depend on the chosen control strategy. For the tree-centered strategy, $p_{1} = \beta _{2}$ and $p_{2} = r_{\tau }$ and; for the vector-centered strategy, $p_{1} = \mu _v$ and $p_{2} = m$.

To achieve this goal, we utilize the difference Eq. (10) and a genetic algorithm to identify the optimal combination of parameter values.

### Algorithm description

A genetic algorithm is a computational optimization technique that is inspired by the process of natural selection and evolution. It involves the use of genetic operators, such as mutation and crossover, to evolve a population of candidate solutions towards an optimal solution. Through successive generations of selection, reproduction, and mutation, genetic algorithms are capable of identifying high-quality solutions to complex optimization problems (Holland 1992).

The genetic algorithm used for this paper involves the following steps:

*Step* 0: Set the non-controlling parameters ($N_{\tau }, \, \mu _{\tau }, \, \sigma$ and $\alpha$) to their baseline values, as shown in Table 1. However, we select the parameter $\beta _{1}$ such that the peak of the asymptomatic curve is less than half the number of citrus trees. Based on the results presented in Sect. (3.2), this value corresponds to $\beta _{1} = 0.0676$. Further, the initial condition for the difference equation (Eq. 10) is defined as $z^{0} = 1$, and the objective value is set to $z^{obj} = \phi N_{\tau }$, where $\phi \in (0,1]$ is a parameter that indicates the desired percentage of roguing trees.

*Step* 1: To begin the genetic algorithm process, we first create a uniform distribution of *M* pairs of control parameter values that fall within the ranges specified in Table 1. If we are using a tree-centered strategy, the parameters to consider are $\beta _{2}$ and $r_{\tau }$, while for a vector-centered strategy, the parameters are *m* and $\mu _{v}$. For each pair of control parameters, we iterate the difference map from Eq. (10) for a specified number of steps (*nstep*) and record the value of the last iteration as $R_{\tau }(\infty )$, which represents the final size of the SAIR-SI model for that specific pair of parameters. Additionally, we evaluate the fitness of each pair of generated parameter values by calculating the absolute difference $| R_{\tau }(\infty ) - z^{obj}|$. Once we have evaluated the fitness of the *M* generated parameter pairs, we obtain the *M*-sized generation 0 of the genetic algorithm.

*Step* 2: (*Elitism*) For each subsequent generation of the genetic algorithm, we select the top-performing individuals from the previous population of size *M*, based on their fitness. Specifically, we choose a fixed percentage $\omega \in [0.1, 1]$ of the population with the best fitness, which are determined by the parameter pairs whose final size approaches the desired objective value, as expressed by $|R_{\tau }(\infty ) - z^{obj}| \le \delta$ with $0< \delta<< 1$. We perform elitism by directly copying the top one pair of parameter, denoted as $p_{1}^{best}$ and $p_{2}^{best}$, to the next generation without any modifications. The remaining individuals that were selected based on their fitness are then recorded in a set $\mathcal {E}$ and subjected to crossover with $p_{1}^{best}$ and $p_{2}^{best}$ to generate a set of $\omega M$ offspring individuals for the subsequent generation, as described in Step 3.

*Step* 3: (*Crossover*) In this step, we use a technique called *Segment Crossover*, also known as *k-point crossover*, to create offspring with unique genetic characteristics from the parent pairs $$p_{1}^{father}$$ and $$p_{2}^{father}$$ (from Step 2, $$p_{i}^{father} = p_{i}^{best}$$, with $$i=1,2$$) and the pair $$p_{1}^{mother}$$ and $$p_{2}^{mother}$$ (if a pair of parameters $$(p_{1},p_{2})$$ is part of the set $$\mathcal {E}$$ in Step 2, then these parameters are assigned as $$p_{1}^{mother}$$ and $$p_{2}^{mother}$$, respectively). The Segment Crossover technique involves randomly selecting at least two crossover points ($$k=2$$) in $$p_{1}^{father}$$ and $$p_{1}^{mother}$$ (similarly for the second parameter by changing sub-index 1 for 2), which are then swapped to produce the offspring’s genetic code. For example, if $$p_{1}^{father} = $$ 0.229**778083**0 and $$p_{1}^{mother} =$$ 0.790**677288**8, where the bold numbers are marked by the two randomly selected crossover points, then, after the crossover operation, the children $$p_{1}^{childs1} =$$ 229**677288**0 and $$p_{1}^{childs2} = $$ 790**778083**8 are generated. The same are performing for the second parameter.

*Step* 4: (*Survival*) This step is used to select the remaining $(1-\omega )M$ individuals for the mating pool after the elite individuals have been selected. Individuals with lower fitness scores, who were not selected for elitism, can be included in the mating pool through this selection process. This process increases the diversity of the population and allows for exploration of the search space beyond local optima. The selection process begins by randomly selecting pairs of individuals from the remaining pool and performing the crossover operation described in Step 3. This process continues until a new generation of *M* individuals is generated.

*Step* 5: (*Mutation*) After creating the offspring generation of *M* individuals, the mutation process randomly selects, for each new individual $p_{1}^{new}$ and $p_{2}^{new}$, one segment (gene) from the descendant individuals, and its value is randomly changed to a value within the range [0, 9]. For example, if $p_{1}^{new}=22960$, the mutation process may change it to $p_{1}^{new}=$ 2**5**960.

*Step* 6: Iterate through steps 2–5 until a specified number of new generations has been produced.

In the following section, we utilize the genetic algorithm outlined earlier to search for the optimal combination of parameters for both tree-centered and vector-centered control strategies. To accomplish this, we begin by generating a population of parameter values within the corresponding ranges provided in Table 1. In each generation, a population of $M=100$ individuals is created and two random crossover points ($k=2$) are selected. To evaluate the final size of the disease for each pair of produced parameter values, we set the parameter $\omega$ to 0.3, which means we select the top 30$\%$ of the population based on their fitness and; a value of $nsteps = 150$ in the difference map equation Eq. (10). This allows us to efficiently assess the final size and optimize the genetic algorithm with minimal iterations, avoiding the need to solve the continuous-time model SAIR-SI.

### Numerical results

**Fig. 7:**
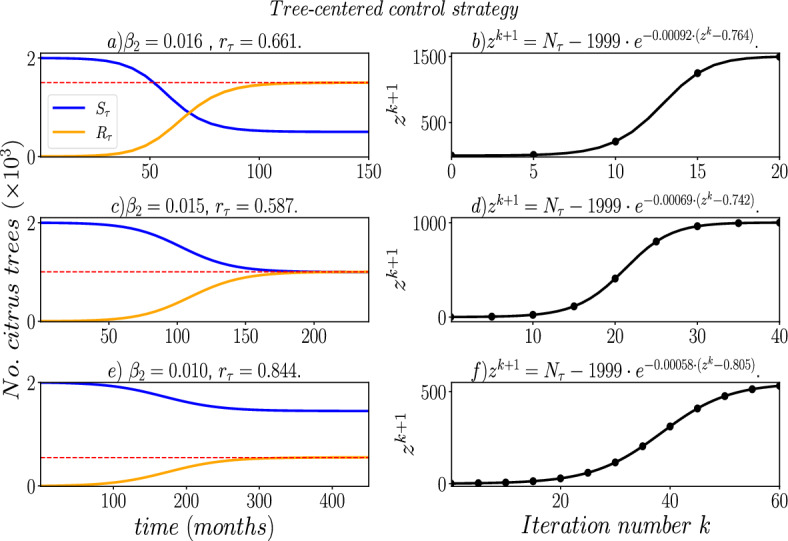
Numerical solution to the SAIR−SI model (Eqs. 1–2) using the parameters $\beta _{2}$ and $r_{\tau }$, which were found using a genetic algorithm, for a tree-centered control strategy. The left subplots display the solution to the difference map (10). The red dashed line marks the target final sizes of $R_{\tau }(\infty ) \rightarrow 0.75 N_{\tau }$, $R_{\tau }(\infty ) \rightarrow 0.5 N_{\tau }$, or $R_{\tau }(\infty ) \rightarrow 0.25 N_{\tau }$, depending on the given percentage of roguing trees (*i.e* $\phi = 0.75, 0.5$ or 0.25 in the Step 0 of the genetic algorithm described in Sect. (5.1))

We utilized the genetic algorithm described previously to find optimal parameter combinations for both tree-centered and vector-centered control strategies. Our objective was to minimize the number of roguing trees at the end of the HLB epidemic in the orchard, which is a reliable indicator of disease severity and control strategy cost. To demonstrate the functionality of the algorithm, we aimed to identify the combination of $\beta _{2}$ and $r_{\tau }$ or $\mu _{v}$ and *m* parameter values for the tree-centered or vector-centered strategy, respectively, that would lead to a final size of the SAIR-SI model reaching $R_{\tau }(\infty ) \rightarrow 0.75 N_{\tau }$, $R_{\tau }(\infty ) \rightarrow 0.5 N_{\tau }$, or $R_{\tau }(\infty ) \rightarrow 0.25 N_{\tau }$, depending on the given percentage of roguing trees (*i.e* $\phi = 0.75, 0.5$ or 0.25 in the Step 0 of the genetic algorithm described in Sect. (5.1)).

In Fig. 7, we can see the SAIR−SI model dynamics for HLB disease in an orchard, using the combination of parameter values $\beta _{2}$ and $r_{\tau }$ obtained from the genetic algorithm. We kept the parameters $\mu _v = 0.5$ and $m = 400.0$ fixed for the tree-centered control. The genetic algorithm identified the values $\beta _{2} = 0.005$ and $r_{\tau }=0.692$ to achieve $R_{\tau }(\infty ) \rightarrow 0.75 N_{\tau }$ (1,500 roguing trees). The red dashed line in Fig. 7 represents the final value of the difference map (10), which was iterated 20 times, yielding $R_{0} = 1.361$. This confirms the predictions from the difference map (10) regarding the final size of the SAIR-SI model.

**Fig. 8:**
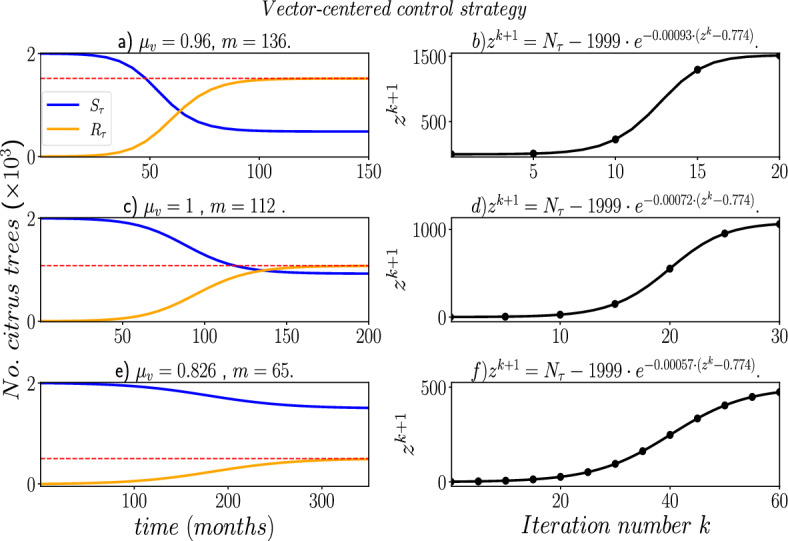
Numerical solution to the SAIR−SI model (Eqs. (1–2) using the parameters $\mu _{v}$ and *m*, which were found using a genetic algorithm, for a vector-centered control strategy. The left subplots display the solution to the difference map (10). The red dashed line marks the target final sizes of $R_{\tau }(\infty ) \rightarrow 0.75 N_{\tau }$, $R_{\tau }(\infty ) \rightarrow 0.5 N_{\tau }$, or $R_{\tau }(\infty ) \rightarrow 0.25 N_{\tau }$, depending on the given percentage of roguing trees (*i.e* $\phi = 0.75, 0.5$ or 0.25 in the Step 0 of the genetic algorithm described in Sect. (5.1))

Similarly, using the genetic algorithm, we found that a combination of $\beta _{2}= 0.003$ and $r_{\tau } = 0.746$ led to $R_{\tau }(\infty ) \rightarrow 0.5 N_{\tau }$, with $R_{0}= 1.154$ Fig. 7d. We also found that setting $\beta _{2}= 0.003$ and $r_{\tau } = 0.873$ resulted in $R_{\tau }(\infty ) \rightarrow 0.25 N_{\tau }$, with $R_{0} = 1.088$ for the difference map, as seen in Fig 7f. However, achieving a 25 % roguing trees requires increased vigilance and several additional months.

It is noteworthy that the values of $\beta _{2}$ obtained using the genetic algorithm fall below the minimum range reported in references and documented in Table 1. This suggests that substantial control measures would be necessary to effectively reduce the transmission rate of HLB from infected trees to susceptible ACP vectors. Such measures could include increasing the deployment of traps designed to capture ACP vectors or implementing physical barriers like fine-mesh nets or screens around the trees. In contrast, to increase the value of the parameter $r_{\tau }$, a systematic program for regularly inspecting trees in the orchard could be established. This program would concentrate on identifying symptoms associated with HLB disease or utilizing advanced diagnostic tools such as PCR, qPCR, or LAMP tests to identify asymptomatic trees.

In contrast, in Fig. 8 presents the outcomes for the vector-centered control strategy, in which we set fixed values of $\beta _{2}=0.0365$ and $r_{\tau }=0.7$, and then explore various combinations of $\mu _{v}$ and *m* to attain a specified percentage *w* of roguing trees by the end of the HLB disease. Specifically, in Fig. 8a, we use the genetic algorithm to determine that $\mu _{v}=0.96$ and $m=136$ lead to 75% of roguing trees, with a corresponding $R_{0}=1.369$, which is confirmed by the dynamics of the difference map in Fig. 8b solved using these parameter values. Note that 20 iterations are needed to achieve the predicted final size $R_{\tau }(\infty )$. Similarly, to attain 50% of roguing trees, we require the parameter combination of $\mu _{v}=1$ and $m=112$, resulting in $R_{0}=1.12$, while for 25% of roguing trees, we need $\mu _{v}=0.826$ and $m=65$, yielding $R_{0}=1.08$.

It is noteworthy that in this particular scenario, achieving a reduction in the number of trees to 25% of the total requires that the maximum abundance of ACP vectors per citrus tree (*m*) be lower than its nominal value as reported in Table 1. This underscores the need for a rigorous vector-centered control strategy aimed at reducing the parameter *m*. Such strategies could involve implementing proper sanitation and pruning techniques or deploying sticky or yellow-colored traps in the orchard to capture and monitor ACP populations. On the other hand, increase the natural mortality rate of ACP ($\mu _{v}$) can involve the targeted application of insecticides specifically designed to affect ACP vectors, or the use of systemic insecticides absorbed by citrus trees and distributed throughout their tissues. When ACP vectors feed on the trees treated with these insecticides, they ingest the chemicals, thereby increasing the mortality rate.

## Conclusions

We have proposed a mathematical model for HLB transmission in a citrus orchard that considers the number of roguing trees and a logistic growth model for the dynamic of the Asian Citrus psyllid (ACP) *Diaphorina citri Kuwayama*, the main responsible for HLB transmission in citrus trees. The Next Generation Matrix methodology was utilized to find an expression for the basic reproduction number, which serves as an indicator for the occurrence of the disease. Through sensitivity analysis PRCC, we determined that the most relevant and influential parameters are $\beta _{2}$, $r_{\tau }$, $\mu _{v}$, and *m*. Based on this information, we defined two control strategies, the tree-centered control and the vector-centered control, which utilize different controlling parameters.

Genetic algorithms, on the other hand, are an efficient way to find optimization parameters in the SAIR-SI model of HLB. This is because genetic algorithms are a type of optimization algorithm that mimic the process of natural selection, and are particularly suited to problems that are difficult to solve using traditional optimization techniques. By using a genetic algorithm based on the expression of the final size of the disease, we were able to efficiently predict, in less of 10 generations, the final size of roguing trees $R(\infty )$ for the proposed SAIR-SI model.

The use of a difference map equation to assess the epidemic final size is an important contribution of the research paper. This is because the difference map is a discrete-time approximation of the continuous-time SAIR-SI model, which reduces the computational burden and allows for a more efficient optimization. By using the difference map equation, we were able to estimate $R(\infty )$ with a small number of iteration steps, rather than solving the continuous-time model SAIR-SI. This not only simplifies the optimization process, but also allows for a faster convergence of the genetic algorithm.

Based on the values obtained with the genetic algorithm, we find that the tree-centered control strategy demands a substantial investment to effectively reduce the transmission rate of HLB from infected trees to susceptible ACP vectors. Additionally, significant efforts are required to enhance vigilance in order to achieve the goal of identifying and rogueing 25% of trees by the end of the epidemic. On the other hand, the vector-centered control strategy requires maintaining the abundance of ACP around 65 per citrus tree to reach half of 25% roguing trees, and around 136 ACP vector to reach 75%, which is costly in terms of more fumigation being required.

Additionally, the findings suggest that some of the optimized parameter values obtained through the genetic algorithm fall outside the typical range reported in the literature. For instance, the values of $\beta _{2}$ obtained for tree-centered control, as well as the parameter *m* for vector-centered control, are notably lower than those reported in the literature (as summarized in Table 1) for a non-controlled orchard. This underscores the necessity for significant control measures to effectively reduce the transmission rate of HLB. Implementing control actions such as increasing trap deployment, frequent fumigation, and employing advanced diagnostic techniques like PCR testing are essential to effectively manage the disease. These strategies should be carefully considered and integrated into comprehensive control programs to mitigate the spread of HLB and protect citrus orchards.

In conclusion, the combination of genetic algorithms and the use of a difference map equation to assess the epidemic final size in the SAIR-SI model of HLB is a powerful optimization tool. This approach allows for the efficient optimization of the model’s parameters and can help in the development of effective strategies for the control and management of the disease. The use of a difference map equation is an important contribution to this research, as it significantly reduces the computational cost and increases the efficiency of the optimization algorithm.

## Appendix A: analytical solution for the final epidemic size using the Lambert W function

In this appendix, we present the explicit analytical solution for the final size of the HLB epidemic using the Lambert W function, based on the previously introduced Eq. (9). The Lambert W function, commonly applied to equations involving products of variables with exponentials, is a powerful tool for solving equations of the form $xe^{x} = z$, which closely resembles the structure of the mathematical expression of the HLB epidemic final size.

Let $x = R_{\tau }(\infty )$, $\displaystyle \gamma =\frac{R_0^2}{N_{\tau }}$, and $\displaystyle \delta = \frac{\mu _{\tau } - r_{\tau }}{\sigma + r_{\tau }}$. Then, Eq. (9) can be rewritten as:

A1 $$\begin{aligned} x = N_{\tau } - S_{\tau }(0) \cdot e^{-\gamma \cdot \delta \cdot I_{\tau }(0)} \cdot e^{-\gamma \cdot x} = N_{\tau } - a \cdot e^{-\gamma \cdot x}, \end{aligned}$$

where $a = S_{\tau }(0) \cdot e^{-\gamma \cdot \delta \cdot I_{\tau }(0)}$. Equation (A1) can be rearranged as:

A2 $$\begin{aligned} (x - N_{\tau }) \cdot e^{\gamma \cdot x} = -a. \end{aligned}$$

By making the substitution $u = \gamma \cdot (x - N_{\tau })$, we obtain:

A3 $$\begin{aligned} \frac{u}{\gamma } \cdot e^{\gamma N_{\tau }} \cdot e^{u} = -a, \end{aligned}$$

which simplifies to:

A4 $$\begin{aligned} u \cdot e^{u} = \frac{-a \cdot \gamma }{e^{\gamma \cdot N_{\tau }}}. \end{aligned}$$

Thus, we can express *u* as:

A5 $$\begin{aligned} u = W\left( -\frac{a \cdot \gamma }{e^{\gamma \cdot N_{\tau }}} \right) , \end{aligned}$$

where $W(\cdot )$ is the Lambert W function. Returning to the original variables, we find the final size $x = R_{\tau }(\infty )$ as:

A6 $$\begin{aligned} x = R_{\tau }(\infty ) = \frac{N_{\tau }}{R_0^2} \cdot W\left( \dfrac{-R_0^2 \cdot S_{\tau }(0)}{N_{\tau }} \cdot e^{-\frac{R_{0}^{2}\cdot \left( N_{\tau } + \delta I_{\tau }(0) \right) }{N_{\tau }} } \right) + N_{\tau }. \end{aligned}$$

Since $R_{\tau }(\infty ) \le N_{\tau }$, it is evident that the argument of the Lambert W function in (A6) must be such that $W(\cdot ) < 0$. However, as noted by Corless et al. (1996), for $x \in \mathbb {R}$, the Lambert W function is only defined for $x \ge -e^{-1}$. When $-e^{-1} \le x < 0$, *W*(*x*) has two possible branches, denoted as $W_{-1}(z)$ and $W_{0}(z)$ Baez-Sanchez and Bobko (2021). In our case, we have the inequality:

$$\begin{aligned} -\frac{1}{e} \le \dfrac{-R_0^2 \cdot S_{\tau }(0)}{N_{\tau }} \cdot e^{-\displaystyle \left( \frac{R_{0}^{2}\cdot \left( N_{\tau } + \delta I_{\tau }(0) \right) }{N_{\tau }} \right) } < 0 \;; \end{aligned}$$

which leads to the conclusion that:

A7 $$\begin{aligned} S_{\tau }(0) \le \dfrac{N_{\tau }}{R^{2}_{0}} \cdot e^{\displaystyle \left( \frac{R_{0}^{2}\cdot \left( N_{\tau } + \delta I_{\tau }(0) \right) - N_{\tau } }{N_{\tau }} \right) } \,. \end{aligned}$$

The inequality (A7) provides a useful bound for the initial susceptible population, $S_{\tau }(0)$, in relation to the basic reproduction number $R_0$ and the initial conditions of the infected population $I_{\tau }(0)$. This result offers a new perspective on the constraints of the system and highlights the relationship between the initial conditions and the epidemic dynamics. Further exploration of this inequality could lead to deeper insights into the optimal allocation of resources for disease control and prevention, especially in cases where early interventions are critical. A thorough investigation of its implications, including numerical validation and potential extensions to more complex models, will be pursued in future work.
